# Supplementary material for: Informal Caregiving in Adolescents from 10 to 16 Years Old: A Longitudinal Study Using Data from the Tokyo Teen Cohort
Source: Int J Environ Res Public Health. 2023 Jul 31;20(15):6482. doi: 10.3390/ijerph20156482 (PMC10419092; doi:10.3390/ijerph20156482)
Supplement: Supplementary file 1 [file ijerph-20-06482-s001.zip › ijerph-2399730-supplementary.pdf]

Supplementary Material

Table S1. Characteristics at 10 years of age between the included 2331 participants and the excluded 840 participants

	Included (n = 2,331)	Excluded (n = 840)[1]	Test statistics	P-value
Sex, N (%)	N = 2,331	N = 840	χ^2 (1) = 0.241	.623
Girl	1,087 (46.6)	400 (47.6)		
Boy	1,244 (53.4)	440 (52.4)		
Number of siblings, N (%)	N = 2,331	N = 840	Mann-Whitney's U test, z = 0.271	.787
None	413 (17.7)	155 (18.5)		
One	1,335 (57.3)	475 (56.5)		
Two or more	583 (25.0)	210 (25.0)		
Single parent, N (%)	N = 2,331	N = 840	χ^2 (1) = 11.714	.001
Yes	99 (4.2)	61 (7.3)		
No	2,232 (95.8)	779 (92.7)		
Low annual household income, less than 400 million yen, N (%)	N = 2,249	N = 797	χ^2 (1) = 5.752	.016
Yes	222 (9.9)	103 (12.9)		
No	2,027 (90.1)	694 (87.1)		
Cohabiting with grandparents, N (%)	N = 2,331	N = 840	χ^2 (1) = 3.092	.079
Yes	192 (8.2)	86 (10.2)		
No	2,139 (91.8)	754 (89.8)		
Daily informal caregiving, N (%)	N = 2,331	N = 835	χ^2 (1) = 0.432	.511
Yes	65 (2.8)	27 (3.2)		

No	2,266 (97.2)	808 (96.8)

[1] Excluded participants comprised 164 drop-put cases at 12 years, 342 at 14 years, and 49 at 16 years of age; and 285 participants at 16 years of age with missing information on informal caregiving.

Table S2. Cross-sectional association between daily informal caregiving and household characteristics at each wave survey with complete cases

	Odds ratio (95% confidence interval) of daily informal caregiving			
	10 years (n = 2,249)	12 years (n = 2,177)	14 years (n = 2,157)	16 years (n = 2,119)
Sex, girl	1.952 (1.152–3.307)*	1.726 (1.044–2.855)*	1.210 (0.738–1.984)	1.718 (1.004–2.941)*
Number of siblings[1]	1.170 (0.870–1.573)	1.286 (0.970–1.705)	1.060 (0.791–1.421)	1.182 (0.870–1.604)
Single parent	0.811 (0.223–2.941)	2.132 (0.832–5.464)	1.624 (0.702–3.755)	1.038 (0.375–2.869)
Low annual household income[2]	1.210 (0.534–2.741)	1.251 (0.538–2.911)	2.954 (1.415–6.170)**	2.013 (0.841–4.820)
Cohabiting with grandparents[3]	4.795 (2.679–8.583)***	5.723 (3.300–9.925)***	7.571 (4.451–12.878)***	8.079 (4.574–14.272)***

Odds ratios and 95% confidence intervals of daily informal caregiving at each wave survey were estimated using multivariate binomial logistic regression analyses.

* significant at $p < .05$; ** $p < .01$; *** $p < .001$.

[1] Number of siblings when the child was 10 years old was used for all analyses.

[2] Less than 400 million yen.

[3] Information on cohabiting with grandparents was not collected at the fourth–wave survey when the child was 16 years old. Thus, cohabiting with grandparents at 14 years of age was used in the analysis of daily informal caregiving at 16 years of age.

Table S3. Longitudinal association between duration of daily informal caregiving from 10 to 16 years of age and household characteristics at 10 years of age with complete cases (n = 2,249).

	Odds ratio (95% confidence interval)	
	Persistent caregiving	Occasional caregiving
Sex, girl	1.783 (0.986–3.223)	1.283 (0.901–1.826)
Number of siblings	1.161 (0.835–1.615)	1.096 (0.883–1.360)
Single parent	1.913 (0.672–5.445)	0.883 (0.368–2.123)
Low annual household income[1]	1.397 (0.596–3.270)	1.301 (0.740–2.284)
Cohabiting with grandparents	8.959 (4.857–16.526)***	4.741 (3.077–7.307)***

Odds ratios and 95% confidence intervals were estimated using multivariate binomial logistic regression analyses with never daily caregiving as a reference group.

* significant at p <.05; ** < .01; *** < .001.

[1] Less than 400 million yen.
